# Non-linear association between life’s essential 8 score and depression in middle-aged and older adults with chronic obstructive pulmonary disease

**DOI:** 10.1371/journal.pone.0327877

**Published:** 2025-07-28

**Authors:** Yushan Shi, Di Huang, Linlin Qu, Qingyin Liu, Zhanjun Qiu, Xianhai Chen

**Affiliations:** 1 Shandong University of Traditional Chinese Medicine, Jinan, China; 2 Department of laboratory, The Affiliated Hospital of Shandong University of Traditional Chinese Medicine, Jinan, China; 3 Department of Respiratory and Critical Care Medicine, The Affiliated Hospital of Shandong University of Traditional Chinese Medicine, Jinan, China; 4 Gerontology Department, The Affiliated Hospital of Shandong University of Traditional Chinese Medicine, Jinan, China; AstraZeneca Pharmaceuticals LP, UNITED STATES OF AMERICA

## Abstract

**Objective:**

Relatively few studies have investigated the link between the Life’s Essential 8 (LE8) score and depression among individuals with Chronic Obstructive Pulmonary Disease (COPD). Our objective was to explore the potential association between the LE8 score and the presence of depression in adults aged ≥40 years who diagnosed with COPD.

**Methods:**

Data from the National Health and Nutrition Examination Survey (NHANES) spanning 2005–2018 was used in this study. Weighted logistic regressions, restricted cubic splines (RCS) were utilized to investigate the correlation between LE8 score and depression. Using a two-piecewise logistic regression model to identify potential threshold effects. Subgroups and sensitivity analyses were conducted to test the robust of the association.

**Results:**

The study encompassed a total of 1,110 subjects diagnosed with COPD. In fully adjusted statistical model, an increment of 10 points in the LE8 score was correlated with a reduced likelihood of depression (OR: 0.68, 95% CI: 0.58–0.78). Similar trends in the associations of health behavior score (OR = 0.75, 95% CI: 0.67–0.84) with depression was also identified. The threshold for the LE8 score was pinpointed at 50.0; surpassing this value, the probability of depression decreased by 8.0% for each additional point in the LE8 score (OR, 0.92; 95% CI: 0.88–0.95; P < 0.001). Furthermore, higher LE8 metric scores of physical activity, nicotine exposure and sleep health were associated with a lower prevalence of depression. The results of subgroup and sensitivity analyses were found to be consistent with the principal analysis.

**Conclusions:**

There is a non-linear relationship between LE8 score and depression, with an inflection point of roughly 50.0. Adhering to a higher LE8 score (≥50.0) was correlated with lower odds of depression among COPD adults aged ≥ 40years.

## Introduction

Chronic obstructive pulmonary disease (COPD) is a leading cause of morbidity and mortality worldwide, characterized by persistent respiratory symptoms and airflow limitation [1,2]. In addition to the physical impact, COPD is also increasingly recognized for its profound psychological burden, with depression being one of the most common comorbidities in this patient population [3]. The coexistence of COPD and depression is clinically significant as it can exacerbate disease severity, impair quality of life, and increase healthcare utilization [4,5]. The analysis demonstrates that patients with comorbid depression have longer hospital stays and a higher risk of mortality post-discharge [6]. Consequently, it is crucial for developing effective preventive strategies that aim to reduce depression prevalence in COPD individuals and alleviate the associated burden.

The concept of Life’s Essential 8 (LE8), as introduced by the American Heart Association, encompasses a set of health behaviors and factors that are pivotal for maintaining cardiovascular health and reducing the risk of cardiovascular diseases [7]. Recent studies have suggested that adherence to the LE8 metrics is not only beneficial for cardiovascular health but may also have a positive impact on other major diseases [8–11]. In addition, several previous studies have estimated the relationship between LE8 and depression [12–14]. A study of 6,851 Chinese participants aged 20 or older found that ideal cardiovascular health was associated with a lower risk of depression, especially among Chinese men and young people [15]. Similar research found that CVH, estimated by the LE8 score, was inversely correlated with the prevalence of depression in US adults [16]. However, the relationship between LE8 and depression, particularly in the context of COPD, remains understudied.

This study aims to fill this gap by investigating the association between LE8 scores and depression in a nationally representative sample of middle-aged and older adults with COPD. This investigation could pave the way for novel therapeutic strategies that integrate lifestyle modifications into the standard treatment of COPD-related depression.

## Materials and methods

### Source of data and study population

This observational study encompassed participants from the NHANES, a nationally representative, serial, and ongoing survey from 2005 to 2018. NHANES is designed to assess the health and nutritional status of the non-institutionalized civilian US population. It employs a multistage, probability sampling design to ensure the sample’s representativeness. Further details on the NHANES methodology are available online (www.cdc.gov/nchs/nhanes/about_nhanes.htm). The study’s procedures were approved by the Ethics Review Board of the National Center for Health Statistics, and all participants provided written informed consent. The research strictly followed the STROBE guidelines for the reporting of observational studies in epidemiology [17]. This study is a secondary analysis of public data, and all participants’ identity information has been anonymized. Consequently, the Ethics Committee of the Affiliated Hospital of Shandong University of Traditional Chinese Medicine has decided to waive the ethical review for this research (NO:2024–0023).

**Fig 1:**
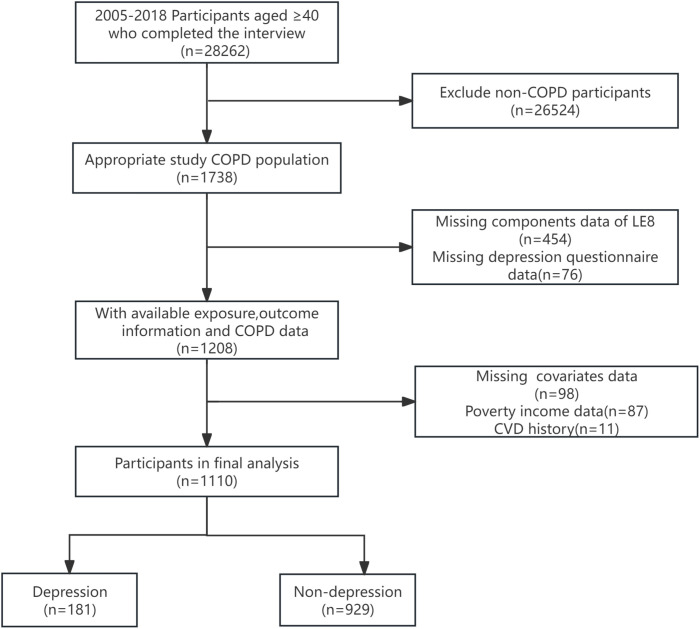
The study’s flow diagram.

A total of 28262 participants aged ≥40 years completed the interview, and 1738 participants were diagnosed with COPD. participants with incomplete data on Life’s Essential 8 (LE8) components (n = 454) and those lacking depression screening data (n = 76) were excluded. Additionally, participants missing key confounder data, such as family income relative to poverty level (n = 87) and history of cardiovascular disease (CVD)(n = 11), were also excluded. The final analytical sample consisted of 1,110 COPD patients, 181 with depression and 929 without depression. Flowchart for participant enrollment is presented in Fig 1.

### Self-reported COPD definition

Referring to previous studies [18,19], COPD is identified by meeting at least one of the following criteria:1. pulmonary function criteria: a FEV1/FVC ratio that is below 0.7 following bronchodilator inhalation. 2. questionnaire responses: affirmative responses to questions regarding a physician’s diagnosis of COPD, emphysema, or chronic bronchitis in the MCQ survey. 3. Pharmacological and Demographic Factors: Individuals over 40 years with a history of smoking or chronic bronchitis who are on therapy with bronchodilators, inhaled steroids, leukotriene modifiers, or phosphodiesterase-4 inhibitors.

### Measurement of LE8 score

The LE8 consists of four health behaviors (diet, physical activity, tobacco/nicotine exposure, and sleep health) and four health factors (body mass index, non-high-density lipoprotein cholesterol, blood glucose, and blood pressure). Each component was scored on a scale from 0 to 100. The composite LE8 score was calculated as the unweighted mean of the individual component scores. Online supplementary materials provide detailed instructions for implementing the LE8 scoring algorithms on the NHANES data for adults (S1 Table) [7,20]. The total LE8 score was classified as high (80–100), moderate (50–79), and low level (0–49).

The Healthy Eating Index 2015 (HEI-2015) was applied to evaluate the dietary intake of participants, based on their first-day 24-hour dietary recall data [21]. The components and scoring criteria for the HEI-2015 are outlined in S2 Table. Physical activity was calculated from self-reported physical activity levels. Tobacco exposure was determined by self-reported smoking status and exposure to secondhand smoke. Sleep duration was evaluated using self-reported average hours of sleep per night. Body mass index (BMI) was calculated by dividing weight (kg) by height squared (m^2^). Blood lipids (non-HDL cholesterol) levels were calculated from fasting blood samples. Blood glucose levels and hemoglobin A1c were assessed from blood samples. To ensure accuracy, blood pressure measurements were derived from the mean of several readings that were recorded throughout the examination process. Medication history was collected by self-report questionnaires.

### Depression assessment

Depression was identified using the Patient Health Questionnaire-9 (PHQ-9) [22]. Study participants were queried about the frequency of their depressive symptoms over the past fortnight. They could respond with one of four options: “not at all” (assigned a score of 0), “on a few days” (assigned a score of 1), “on more than half the days” (assigned a score of 2), or “nearly every day” (assigned a score of 3). The scores for the nine items were tallied to compute a total score for each individual, which spanned from 0 to 27. A threshold of 10 or above on the PHQ-9 was used to diagnose the presence of depressive disorders [23].

### Assessment of covariates

Trained interviewers collected demographic and laboratory information including age, sex (female and male), race and ethnicity (Mexican American, Non-Hispanic White, Non-Hispanic Black, Other Hispanic, and other race/multiracial), family income, marry status, education level, urinary albumin-to-creatinine ratio (UACR), estimate glomerular filtration rate (eGFR), history of cardiovascular disease (CVD), and chronic kidney disease (CKD). Family income was defined using the poverty income ratio (PIR) and then classified into 3 groups: Low income ≤1.30, moderate income (1.31 to 3.50), and high income (>3.50) [24]. Marital status was as living with a partner, married, never married, and others. Educational attainment was stratified into three categories: below high school, high school, and beyond high school. Participants self-reported their history of cardiovascular diseases (CVD), which included previous diagnoses of heart failure, coronary artery disease, angina, myocardial infarction, or stroke [25]. The estimated glomerular filtration rate (eGFR) was determined using the Chronic Kidney Disease Epidemiology Collaboration (CKD-EPI) equation, which is based on serum creatinine levels [26]. Albuminuria was assessed by calculating the ratio of urinary albumin to creatinine. Chronic kidney disease (CKD) was diagnosed if the eGFR was less than <60mL/min per 1.73m2, albuminuria was 30 mg/g or higher, or if both criteria were met [27].

### Statistical analysis

Statistical analyses were all computed using sampling weights to guarantee that the results are representative at a national level. Participant demographics at baseline were described based on their LE8 scores. For continuous variables, the mean and standard deviation [SD] were calculated and reported. Categorical variables were expressed in terms of the number of participants in millions along with their respective percentage frequencies (%). To identify significant differences among various levels of cardiovascular health (CVH), a chi-squared (χ2) test was conducted for categorical data, while an analysis of variance (ANOVA) was applied to the continuous data sets.

A multivariable logistic regression analysis was performed to explore the relationship between LE8 scores, health factor score, the health behaviors score, 8 LE8 metrics and the prevalence of depressive. LE8 score was entered as a continuous variable (per 10 points increase) and as a categorical variable (low, moderate, and high level). Crude model was not adjusted; Model 1 was adjusted for age, sex, race/ethnicity; Model2 was adjusted for age, sex, race/ethnicity, marital status, educational level, PIR, CVD history, and CKD history.

To assess the non-linear association between LE8 scores and depression, we employed restricted cubic splines (RCS) in a logistic regression model. A two-piecewise logistic regression model was employed to identify potential threshold effects.

To delve deeper into how the LE8 score correlates with depression across various demographic groups, stratified analyses were conducted by age (<65years or ≥65 years), sex (female or male), poverty (≤1.30, 1.31–3.50, or ≥3.50), education levels (less than high school, high school, or above high school), and history of CVD (yes or no), CKD (yes or no). The significance of interactions was estimated using *P* values for the interaction coefficients between LE8 score and subgroup populations.

To substantiate the validity of our findings, a variety of sensitivity analyses were performed. We reiterated the primary analyses to assess the relationship between the quartiles of the Life’s Essential 8 (LE8) score and the occurrence of depression. Additionally, we conducted unweighted logistic regression analyses to further evaluate this association. Furthermore, we employed multiple imputation methods to account for missing data on covariates.

All statistical analyses were conducted using R version 4.3.2 (The R Foundation, http://www.R-project.org), and the Free Statistics software version 2.0. A *P*-value less than 0.05 (two-tailed) was considered to indicate statistical significance.

## Result

### Characteristics of the participants

Table 1 illustrates the baseline demographics of the 1,110 individuals with complete data suitable for analysis, which corresponds to an estimated 6.64 million US adults aged 40 years and older who have COPD. The weighted mean age was 61.66 years and 49.88% of the participants were female. Participants in the high CVH group were typically non-Hispanic white, married, wealthier, educated above high school, and had no history of CVD, depression, and CKD.

**Table 1:** Baseline characteristics by cardiovascular health score level.

Variables	Total	Low CVH (LE8 < 50)	Moderate CVH (50 ≤ LE8 < 80)	High CVH (LE8 ≥ 80)	*P*
Weighted population, n [in millions]	6.64	1.66	4.57	0.41	
Age, year	61.66 (10.90)	61.13 (10.73)	61.63 (11.00)	64.21 (10.33)	0.17
Sex, n [in millions] (%)					
Female	3.31 (49.88)	0.82(49.51)	2.21 (48.27)	0.28 (69.21)	0.03
Male	3.33 (50.12)	0.84 (50.49)	2.36(51.73)	0.13(30.79)	
Race/ethnicity, *n* [in millions] (%)					
Mexican American	0.10(1.60)	0.03(2.05)	0.07(1.51)	0.003 (0.74)	<0.001
Non-Hispanic Black	0.40(6.06)	0.16(10.19)	0.22(4.84)	0.01(3.06)	
Non-Hispanic White	5.56 (83.76)	1.35(81.67)	3.82 (83.59)	0.38(94.04)	
Other Hispanic	0.14 (2.12)	0.03(1.92)	0.10(2.35)	0.001(0.32)	
Other Race – Including Multi-Racial	0.42(6.46)	0.06(4.17)	0.35(7.71)	0.007(1.83)	
Marital status, *n* [in millions] (%)					
Living with partner	0.37(5.61)	0.15(9.61)	0.21 (4.52)	0.01(1.49)	<0.001
Married	3.99(60.13)	0.81(48.88)	2.86 (62.68)	0.32(77.08)	
Never married	0.28(4.30)	0.08 (4.93)	0.19(4.27)	0.01(2.15)	
others	1.99(29.96)	0.60(36.58)	1.31(28.53)	0.08(19.28)	
PIR, n [in millions] (%)					
Low income (≤1.30)	1.51 (22.86)	0.63 (38.13)	0.86 (18.98)	0.02(4.38)	<0.001
Moderate income (1.31–3.50)	2.48 (37.38)	0.68 (41.28)	1.65 (36.20)	0.15(34.85)	
High income (>3.50)	2.64 (39.76)	0.34 (20.59)	2.04 (44.82)	0.25(60.77)	
Education, *n* [in millions] (%)					
High School	1.75(26.45)	0.46(27.75)	1.23(26.85)	0.06(16.75)	<0.001
Less Than high school	1.23(18.60)	0.48(29.19)	0.72(15.77)	0.03(7.29)	
More than high school	3.64(54.95)	0.71(43.05)	2.62(57.38)	0.31(75.96)	
eGFR, ml/ (min·1.73m^2^)	80.01 (19.30)	79.707(22.13)	80.10(18.36)	80.15 (17.42)	0.96
UACR, mg/g	36.95(199.764)	58.75 (235.686)	31.66 (194.155)	9.09 (10.069)	<0.001
CVD, n [in millions] (%)					
No	4.85(72.96)	0.98(58.79)	3.53(77.25)	0.34(82.33)	<0.001
Yes	1.79(27.04)	0.68(41.21)	1.04 (22.75)	0.07(17.67)	
CKD, n [in millions] (%)					
No	5.08(76.58)	1.12(67.55)	3.61 (78.95)	0.35 (86.70)	<0.001
Yes	1.56(23.42)	0.54(32.45)	0.96(21.05)	0.05(13.30)	
Depression, n [in millions] (%)					
No	5.80(87.26)	1.28(77.31)	4.11(89.94)	0.41(97.55)	<0.001
Yes	0.84(12.74)	0.37(22.69)	0.46(10.06)	0.01(2.45)	
AHA LE8 scores					
Total CVH score	59.91 (13.97)	41.29 (6.43)	64.40 (7.95)	85.15 (4.48)	<0.001
Health behaviors score	57.30 (20.65)	36.54 (14.19)	62.16 (16.81)	86.97 (7.15)	<0.001
Diet score	38.91 (31.20)	20.30 (22.22)	42.46 (30.74)	74.45 (20.92)	<0.001
Physical activity score	61.20 (45.68)	26.82 (41.66)	70.28 (41.95)	98.83 (7.42)	<0.001
Nicotine exposure score	50.52 (39.31)	31.81 (36.11)	54.37 (38.87)	83.20 (16.87)	<0.001
Sleep health score	78.56 (28.11)	67.23 (31.93)	81.52 (26.25)	91.41 (14.85)	<0.001
Health factors score	62.53 (17.54)	46.03 (14.26)	66.64 (14.60)	83.34 (7.92)	<0.001
BMI score	57.56 (34.04)	34.97 (31.09)	63.36 (32.08)	84.14 (15.96)	<0.001
Blood lipid score	58.61(29.57)	44.65 (30.29)	61.81 (27.84)	79.33 (22.29)	<0.001
Blood glucose score	75.53 (27.62)	57.18 (28.04)	80.15 (25.06)	98.20 (8.34)	<0.001
Blood pressure score	58.41 (30.26)	47.34 (31.03)	61.23 (29.57)	71.68 (21.07)	<0.001

**Abbreviations:** CVH, cardiovascular health; LE8, Life’s Essential 8; PIR, poverty income ratio; eGFR, estimated glomerular filtration rate;

UACR, urinary albumin to creatinine ratio; CVD, cardiovascular disease; CKD, chronic kidney disease; AHA, American heart association; BMI, body mass index.

### Association of LE8 score and LE8 components with depression

The relationship between the LE8 score with depression is detailed in Table 2. An increase of 10 points in the LE8 score was linked to a reduced risk of depression (OR: 0.68, 95% CI: 0.58–0.78) after controlling for various covariates in the second model. When categorized, individuals with moderate and high LE8 scores showed a lower likelihood of depression compared to those with low scores (moderate: OR: 0.59, 95% CI: 0.37–0.94; high: OR: 0.22, 95% CI: 0.05–0.94) in model 2. Similarly, an increase of 10 points in the health behaviors score was linked to a reduced risk of depression (OR: 0.75, 95% CI: 0.67–0.84). Furthermore, higher LE8 metric scores of physical activity (OR: 0.94, 95% CI: 0.89–0.99), nicotine exposure (OR: 0.93, 95% CI: 0.88–0.97). and sleep health (OR: 0.88, 95% CI: 0.82–0.95) were associated with a lower prevalence of depression.

**Table 2:** The associations between the LE8 score, health factor score, the health behaviors score, 8 LE8 metrics and depression.

Variable	Crude model		Model 1		Model 2	
	OR (95%CI)	*p*	OR (95%CI)	*p*	OR (95%CI)	*p*
**Total LE8 score**						
Low (0–49)	Reference		Reference		Reference	
Moderate (50–79)	0.38(0.25-0.58)	<0.001	0.38(0.25-0.58)	<0.001	0.59(0.37-0.94)	0.027
High (80–100)	0.09(0.02-0.32)	<0.001	0.09(0.02-0.35)	<0.001	0.22(0.05-0.94)	0.041
*p* for trend		<0.001		<0.00		0.009
Per 10-points increase	0.57(0.51-0.65)	<0.001	0.57(0.51-0.65)	<0.001	0.68(0.58-0.78)	<0.001
**Health behaviors score**						
Low (0–49)	Reference		Reference		Reference	
Moderate (50–79)	0.34(0.22-0.54)	<0.001	0.34(0.21-0.54)	<0.001	0.46(0.26-0.80)	0.006
High (80–100)	0.13(0.06-0.29)	<0.001	0.14(0.06-0.29)	<0.001	0.26(0.12-0.60)	0.002
*p* for trend		<0.001		<0.001		<0.001
Per 10-points increase	0.66(0.60-0.72)	<0.001	0.67(0.61-0.73)	<0.001	0.75(0.67-0.84)	<0.001
**Health factors score**						
Low (0–49)	Reference		Reference		Reference	
Moderate (50–79)	0.82(0.47-1.40)	0.46	0.85(0.48-1.49)	0.564	1.18(0.61-2.28)	0.626
High (80–100)	0.55(0.28-1.07)	0.076	0.47(0.23-0.97)	0.041	0.73(0.35-1.51)	0.395
*p* for trend		0.093		0.052		0.544
Per 10-points increase	0.87(0.77-0.99)	0.03	0.85(0.76-0.96)	0.009	0.93(0.83-1.05)	0.237
**8 LE8 metrics** (Per 10-point increase)						
HEI-2015 diet score	0.89(0.84-0.94)	<0.001	0.90(0.85-0.96)	0.002	0.95(0.89-1.02)	0.146
Physical activity score	0.92(0.88-0.96)	<0.001	0.91(0.86-0.95)	<0.001	0.94(0.89-0.99)	0.023
Nicotine exposure score	0.86(0.82-0.91)	<0.001	0.88(0.84-0.92)	<0.001	0.93(0.88-0.97)	0.004
Sleep health score	0.83(0.79-0.89)	<0.001	0.84(0.79-0.90)	<0.001	0.88(0.82-0.95)	<0.001
Body mass index score	0.93(0.87-1.00)	0.063	0.94(0.88-1.01)	0.087	0.97(0.91-1.04)	0.435
Blood lipids score	0.96(0.89-1.04)	0.284	0.97(0.90-1.05)	0.484	0.98(0.90-1.07)	0.656
Blood glucose score	0.96(0.89-1.03)	0.261	0.94(0.87-1.01)	0.083	0.99(0.92-1.07)	0.854
Blood pressure score	0.98(0.91-1.06)	0.605	0.94(0.86-1.02)	0.155	0.96(0.87-1.06)	0.42

**Abbreviations:** CI, confidence interval; OR, odds ratio; PIR, poverty income ratio, CVD, cardiovascular disease; CKD, chronic kidney disease.

Crude model: unadjusted.

Model 1: adjusted for age, sex, race/ethnicity.

Model2: adjusted for age, sex, race/ethnicity, marital status, educational level, PIR, CVD history, and CKD history.

### Nonlinear relationship betweenLE8 score and the prevalence of depression

**Fig 2:**
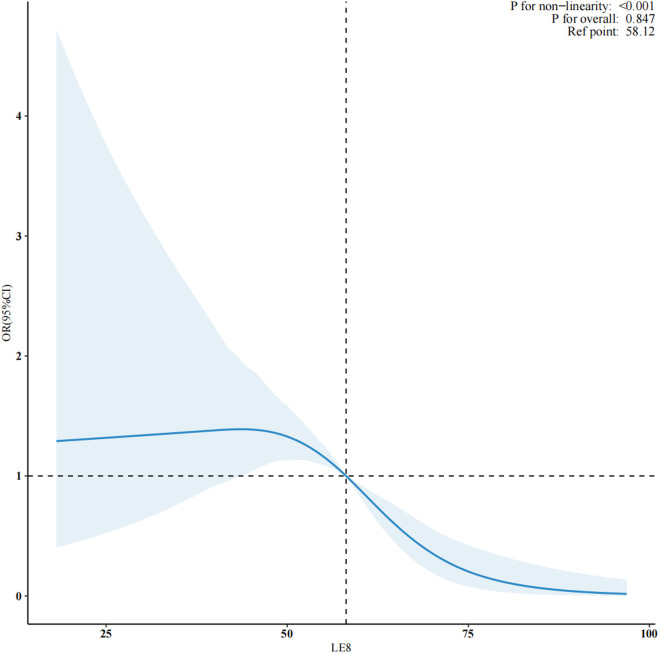
Association between LE8 score and depression odds ratio. Solid and dashed lines represent the predicted value and 95% confidence intervals. They were adjusted for age, sex, race/ethnicity, marital status, educational level, poverty income ratio, cardiovascular disease, chronic kidney disease.

After accounting for certain covariates in model 2, we identified a nonlinear dose-response (*P* for nonlinear <0.001) relationship between the LE8 score and depression (Fig 2). Using a two-piecewise logistic regression model, the LE8 score threshold was determined to be 50.0 (Table 3). Below this threshold, there was no significant association between the LE8 score and depression (OR, 0.97; 95% CI: 0.93–1.02; P = 0.21). Conversely, above this threshold, the risk of depression is reduced by 8.0% with each unit increase in LE8 score (OR, 0.92; 95% CI: 0.88–0.95; P < 0.001).

**Table 3:** Threshold effect analysis of the relationship of LE8 with depression.

Threshold of LE8 score	OR (95%CI)	*p*
<50.0	0.97(0.93-1.02)	0.21
≥50.0	0.92(0.88-0.95)	<0.001

**Abbreviations:** CI, confidence interval; OR, odds ratio; PIR, poverty income ratio, CVD, cardiovascular disease; CKD, chronic kidney disease.

Adjusted for age, sex, race/ethnicity, marital status, educational level, PIR, CVD history, and CKD history.

### Subgroup analyses

**Fig 3:**
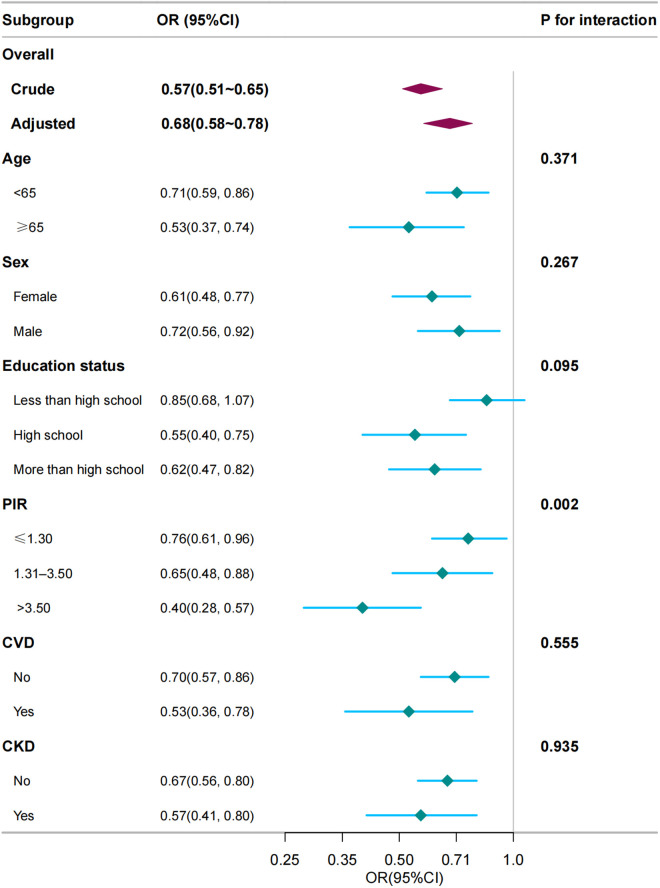
The relationship between LE8 score and depression according to basic features. Except for the stratification component itself, each stratification factor was adjusted for age, sex, race/ethnicity, marital status, educational level, poverty income ratio, cardiovascular disease, chronic kidney disease.

The findings of the subgroup analyses are shown in Fig 3. Upon stratification by age (<65years or ≥65 years), sex (female or male), poverty (≤1.30, 1.31–3.50, or ≥3.50), education levels (less than high school, high school, or above high school) and history of CVD (yes or no), CKD (yes or no). no significant interactions were observed among the subgroups except PIR group.

### Sensitivity analysis

The correlation of elevated LE8 scores with a reduced likelihood of depression was consistently significant in all sensitivity analyses. After categorizing the participants based on the quartiles of their LE8 scores, it was observed that higher quartiles correlated with a decreased risk of depression, as detailed in S3 Table of the Supplementary material. The findings from the unweighted logistic regression analysis were consistent with the primary analysis, as shown in S4 Table of the Supplementary material. Additionally, the consistency of the results was upheld following the application of multiple imputation methods to account for missing data in covariates, with the results presented in S5 Table of the Supplementary material.

## Discussion

In our study, we observed a significant nonlinear association between LE8 scores and depressive among middle-aged and older adults with COPD. As the LE8 score increases, the prevalence of depression decreases, but this relationship is most pronounced when the LE8 score exceeds a certain threshold (50.0). Furthermore, higher LE8 metric scores of physical activity, nicotine exposure and sleep health were associated with a lower prevalence of depression. Multiple sensitivity and stratified analyses further confirmed the robustness of these findings, suggesting that increasing LE8 score could potentially reduce depression risk in individuals with COPD.

In extensive longitudinal cohort research involving participants without cardiovascular disease (CVD) or prevalent mental health disorders, a higher Life’s Essential 8 (LE8) score correlates with a lower likelihood of developing depression [14]. In the United Kingdom, among individuals aged 50 and above, higher scores were linked to a reduced risk of depressive conditions [28]. Studies have also shown that a higher baseline LE8 score is associated with a decreased risk of depressive symptoms in the general population [29,30]. Numerous cross-sectional investigations have reported an inverse relationship between the LE8 score and depression among US adults [12,16,31]. This resonates with our finding that higher LE8 scores are associated with a reduced risk of depressive symptoms. Most existing studies have focused on the general adult population, whereas our study concentrates on middle-aged and older adults with COPD. Further, our study indicated that LE8 was non-linear inversely associated with depression, and this has not been fully explored in previous research. This study shown that the association between the LE8 score and depression becomes more pronounced after exceeding a specific threshold, which may imply that for COPD patients, maintaining a certain level of cardiovascular health behaviors and factors could have additional importance in preventing depression. As known, COPD patients often experience dyspnea, decreased exercise tolerance, and frequent acute exacerbations, all of which can exacerbate psychological distress [32]. The likelihood of a COPD patient developing comorbid depression is four times higher than that of an individual without COPD [33]. In addition, COPD patients often require long-term use of bronchodilators, inhaled corticosteroids, and other medications, which may affect cardiovascular health and psychological status, thereby indirectly affecting the relationship between LE8 scores and depression. Consequently, the relationship between LE8 scores and depressive symptoms in COPD patients may be more complex compared to the general population. Furthermore, we employed multivariate logistic regression models to examine the relationships between all 8 LE8 components and COPD. The results suggested that apart from physical activity (*P*-value < 0.05), nicotine exposure (*P*-value < 0.01) and sleep health metrics (*P*-value < 0.001) in LE8, the effects of the other LE8 components on COPD were relatively limited (P-value > 0.05). Previous studies have shown that poor sleep quality is significantly associated with severe COPD [34]. This may further aggravate the occurrence of depression. It can be seen that solving sleep problems in patients with chronic obstructive pulmonary disease may alleviate depression.

Furthermore, results of subgroup analyses showed the relationship between LE8 score and depressive is particularly significant among people with higher family income. Growing evidence suggested the association between economic inequality and mental disorders [35,36] and it has been reported that lower socioeconomic status were related to higher risk of developing depression [37,38]. This study may suggest that in COPD patients, lower household income could intensify the psychological burden caused by the disease, thereby increasing the risk of depressive. This finding suggests that when providing care for COPD patients, special attention should be given to those with lower socioeconomic status, and targeted interventions should be considered to alleviate their psychological burden. Further research is needed in the future to explore the heterogeneity of the relationship between LE8 scores and depression among COPD patients from different socioeconomic backgrounds.

Our research offers several notable strengths. It pioneers the exploration of the link between the LE8 score and the onset of depressive within the demographic affected by COPD. To ascertain the robustness of the observed association, we executed a series of subgroup analyses and sensitivity assessments. These methodologies fortified our conclusions by confirming the stability of the relationship under various conditions. The cross-sectional design of our study precludes the drawing of causal conclusions. Additionally, while we utilized sampling weights to enhance the precision of our data, the reliance on self-reported questionnaires makes recall bias a potential concern. It is also possible that there were unmeasured confounding variables that we could not account for in our analyses, despite our adjustment for several known confounders. Furthermore, since our study focused on COPD patients aged 40 years and older, the applicability of our findings to younger populations with COPD may be limited. Therefore, additional prospective research is warranted to confirm the potential preventative effects of LE8 on depression in this specific population.

## Conclusion

A non-linear relationship between LE8 score and depression risk was found in patients with COPD. The risk of depression decreased by 8.0% for every one unit increase in LE8 score when LE8 score≥50.0. Furthermore, higher LE8 metric scores of physical activity, nicotine exposure and sleep health were associated with a lower prevalence of depression. Future research should focus on exploring the precise mechanisms underlying the association between le8 and depression, and consider the role of socioeconomic factors in it.

## Supporting information

S1 Table Definition and scoring approach for the American Heart Association’s Life’s Essential 8 score. (DOCX)

S2 Table Healthy Eating Index-2015 Components & Scoring Standards. (DOCX)

S3 Table Association between Life’s Essential 8 score and depression by quartile. (DOCX)

S4 Table Unweighted logistic analysis of association between Life’s Essential 8 score and depression. (DOCX)

S5 Table The relationship between LE8 and depression after imputation of missing values. (DOCX)

## References

[1] Christenson SA, Smith BM, Bafadhel M, Putcha N. Chronic obstructive pulmonary disease. Lancet. 2022;399(10342):2227–42. doi: 10.1016/S0140-6736(22)00470-6 35533707

[2] Global incidence, prevalence, years lived with disability (YLDs), disability-adjusted life-years (DALYs), and healthy life expectancy (HALE) for 371 diseases and injuries in 204 countries and territories and 811 subnational locations, 1990-2021: a systematic analysis for the Global Burden of Disease Study 2021. Lancet. 2024;403(10440):2133–61. 38642570 10.1016/S0140-6736(24)00757-8 PMC11122111

[3] Garvey C, Criner GJ. Impact of Comorbidities on the Treatment of Chronic Obstructive Pulmonary Disease. Am J Med. 2018;131(9S):23–9. doi: 10.1016/j.amjmed.2018.05.002 29777661

[4] Yohannes AM, Alexopoulos GS. Depression and anxiety in patients with COPD. Eur Respir Rev. 2014;23(133):345–9. doi: 10.1183/09059180.00007813 25176970 PMC4523084

[5] Maurer J, Rebbapragada V, Borson S, Goldstein R, Kunik ME, Yohannes AM, et al. Anxiety and depression in COPD: current understanding, unanswered questions, and research needs. Chest. 2008;134(4 Suppl):43S-56S. doi: 10.1378/chest.08-0342 18842932 PMC2849676

[6] Mao W, Shalaby R, Owusu E, Elgendy HE, Agyapong B, Eboreime E, et al. Depression, anxiety, and poor well-being at discharge from psychiatric hospitals: prevalence and risk factors. Front Psychiatry. 2024;15:1408095. doi: 10.3389/fpsyt.2024.1408095 39056021 PMC11269243

[7] Lloyd-Jones DM, Allen NB, Anderson CAM, Black T, Brewer LC, Foraker RE, et al. Life’s Essential 8: Updating and Enhancing the American Heart Association’s Construct of Cardiovascular Health: A Presidential Advisory From the American Heart Association. Circulation. 2022;146(5):e18–43. doi: 10.1161/CIR.0000000000001078 35766027 PMC10503546

[8] Petermann-Rocha F, Deo S, Celis-Morales C, Ho FK, Bahuguna P, McAllister D, et al. An Opportunity for Prevention: Associations Between the Life’s Essential 8 Score and Cardiovascular Incidence Using Prospective Data from UK Biobank. Curr Probl Cardiol. 2023;48(4):101540. doi: 10.1016/j.cpcardiol.2022.101540 36528209

[9] Wang X, Ma H, Li X, Heianza Y, Manson JE, Franco OH, et al. Association of Cardiovascular Health With Life Expectancy Free of Cardiovascular Disease, Diabetes, Cancer, and Dementia in UK Adults. JAMA Intern Med. 2023;183(4):340–9. doi: 10.1001/jamainternmed.2023.0015 36848126 PMC9972243

[10] Wang L, Yi J, Guo X, Ren X. Associations between life’s essential 8 and non-alcoholic fatty liver disease among US adults. J Transl Med. 2022;20(1):616. doi: 10.1186/s12967-022-03839-0 36564799 PMC9789599

[11] Ren Y, et al. Associations between life’s essential 8 and chronic kidney disease. J Am Heart Assoc. 2023;12(24):e030564. 10.1161/JAHA.123.030564 PMC10863789 38063194

[12] Shen R, Zou T. The association between cardiovascular health and depression: Results from the 2007-2020 NHANES. Psychiatry Res. 2024;331:115663. doi: 10.1016/j.psychres.2023.115663 38064908

[13] Li L, Dai F. Comparison of the associations between Life’s Essential 8 and Life’s Simple 7 with depression, as well as the mediating role of oxidative stress factors and inflammation: NHANES 2005-2018. J Affect Disord. 2024;351:31–9. doi: 10.1016/j.jad.2024.01.200 38280569

[14] Huang X, Zhang J, Liang J, Duan Y, Xie W, Zheng F. Association of Cardiovascular Health With Risk of Incident Depression and Anxiety. Am J Geriatr Psychiatry. 2024;32(5):539–49. doi: 10.1016/j.jagp.2023.10.017 37968161

[15] Li Z, Yang X, Wang A, Qiu J, Wang W, Song Q, et al. Association between Ideal Cardiovascular Health Metrics and Depression in Chinese Population: A Cross-sectional Study. Sci Rep. 2015;5:11564. doi: 10.1038/srep11564 26176196 PMC4648472

[16] Zeng G, Lin Y, Lin J, He Y, Wei J. Association of cardiovascular health using Life’s Essential 8 with depression: Findings from NHANES 2007-2018. Gen Hosp Psychiatry. 2024;87:60–7. doi: 10.1016/j.genhosppsych.2024.01.011 38306947

[17] von Elm E, Altman DG, Egger M, Pocock SJ, Gøtzsche PC, Vandenbroucke JP, et al. The Strengthening the Reporting of Observational Studies in Epidemiology (STROBE) statement: guidelines for reporting observational studies. Lancet. 2007;370(9596):1453–7. doi: 10.1016/S0140-6736(07)61602-X 18064739

[18] Han L, Wang Q. Associations of brominated flame retardants exposure with chronic obstructive pulmonary disease: A US population-based cross-sectional analysis. Front Public Health. 2023;11:1138811. doi: 10.3389/fpubh.2023.1138811 36969665 PMC10036799

[19] Xiao Y, Zhang L, Liu H, Huang W. Systemic inflammation mediates environmental polycyclic aromatic hydrocarbons to increase chronic obstructive pulmonary disease risk in United States adults: a cross-sectional NHANES study. Front Public Health. 2023;11:1248812. doi: 10.3389/fpubh.2023.1248812 38074734 PMC10703366

[20] Lloyd-Jones DM, Ning H, Labarthe D, Brewer L, Sharma G, Rosamond W, et al. Status of Cardiovascular Health in US Adults and Children Using the American Heart Association’s New “Life’s Essential 8” Metrics: Prevalence Estimates From the National Health and Nutrition Examination Survey (NHANES), 2013 Through 2018. Circulation. 2022;146(11):822–35. doi: 10.1161/CIRCULATIONAHA.122.060911 35766033

[21] Krebs-Smith SM. Update of the Healthy Eating Index: HEI-2015. J Acad Nutr Diet. 2018;118(9):1591–602. 30146071 10.1016/j.jand.2018.05.021 PMC6719291

[22] Ballou S. Chronic diarrhea and constipation are more common in depressed individuals. Clin Gastroenterol Hepatol. 2019;17(13):2696–703. 30954714 10.1016/j.cgh.2019.03.046 PMC6776710

[23] Kroenke K, Spitzer RL, Williams JB. The PHQ-9: validity of a brief depression severity measure. J Gen Intern Med. 2001;16(9):606–13. doi: 10.1046/j.1525-1497.2001.016009606.x 11556941 PMC1495268

[24] Tian S, Wu L, Zheng H, Zhong X, Liu M, Yu X, et al. Dietary niacin intake in relation to depression among adults: a population-based study. BMC Psychiatry. 2023;23(1):678. doi: 10.1186/s12888-023-05188-8 37723526 PMC10506255

[25] Zhang Y-B, Chen C, Pan X-F, Guo J, Li Y, Franco OH, et al. Associations of healthy lifestyle and socioeconomic status with mortality and incident cardiovascular disease: two prospective cohort studies. BMJ. 2021;373:n604. doi: 10.1136/bmj.n604 33853828 PMC8044922

[26] Levey AS, Stevens LA, Schmid CH, Zhang YL, Castro AF 3rd, Feldman HI, et al. A new equation to estimate glomerular filtration rate. Ann Intern Med. 2009;150(9):604–12. doi: 10.7326/0003-4819-150-9-200905050-00006 19414839 PMC2763564

[27] Kidney Disease: Improving Global Outcomes (KDIGO) Glomerular Diseases Work Group. KDIGO 2021 Clinical Practice Guideline for the Management of Glomerular Diseases. Kidney Int. 2021;100(4S):S1–276. doi: 10.1016/j.kint.2021.05.021 34556256

[28] Gao B, Song S, Guo J. Associations between life’s simple 7 and incident depression among adults aged 50 years and older: A 15-year cohort study. Psychiatry Res. 2023;320:115046. doi: 10.1016/j.psychres.2022.115046 36599180

[29] Adams S. Vascular risk factor burden and new-onset depression in the community. Prev Med. 2018;111:348–50. 29197532 10.1016/j.ypmed.2017.11.022 PMC5930122

[30] España-Romero V, Artero EG, Lee D-C, Sui X, Baruth M, Ruiz JR, et al. A prospective study of ideal cardiovascular health and depressive symptoms. Psychosomatics. 2013;54(6):525–35. doi: 10.1016/j.psym.2013.06.016 24012292

[31] Zhao S, Tang Y, Li Y, Shen H, Liu A. Associations between Life’s Essential 8 and depression among US adults. Psychiatry Res. 2024;338:115986. doi: 10.1016/j.psychres.2024.115986 38850892

[32] Hegerl U, Mergl R. Depression and suicidality in COPD: understandable reaction or independent disorders?. Eur Respir J. 2014;44(3):734–43. doi: 10.1183/09031936.00193213 24876171

[33] Bratek A, Zawada K, Beil-Gawełczyk J, Beil S, Sozańska E, Krysta K, et al. Depressiveness, symptoms of anxiety and cognitive dysfunctions in patients with asthma and chronic obstructive pulmonary disease (COPD): possible associations with inflammation markers: a pilot study. J Neural Transm (Vienna). 2015;122 Suppl 1(Suppl 1):S83-91. doi: 10.1007/s00702-014-1171-9 24532256 PMC4529448

[34] Clímaco DCS, Lustosa TC, Silva MV de FP, Lins-Filho OL, Rodrigues VK, Oliveira-Neto L de AP de, et al. Sleep quality in COPD patients: correlation with disease severity and health status. J Bras Pneumol. 2022;48(3):e20210340. doi: 10.36416/1806-3756/e20210340 35508063 PMC9064624

[35] Gero K, Yazawa A, Kondo N, Hanazato M, Kondo K, Kawachi I. Comparison of three indices of relative income deprivation in predicting health status. Soc Sci Med. 2022;294:114722. doi: 10.1016/j.socscimed.2022.114722 35065345 PMC8844705

[36] Linder A, Gerdtham U-G, Trygg N, Fritzell S, Saha S. Inequalities in the economic consequences of depression and anxiety in Europe: a systematic scoping review. Eur J Public Health. 2020;30(4):767–77. doi: 10.1093/eurpub/ckz127 31302703 PMC7445046

[37] Qi X, Jia Y, Pan C, Li C, Wen Y, Hao J, et al. Index of multiple deprivation contributed to common psychiatric disorders: A systematic review and comprehensive analysis. Neurosci Biobehav Rev. 2022;140:104806. doi: 10.1016/j.neubiorev.2022.104806 35926729

[38] Ye J, Wen Y, Sun X, Chu X, Li P, Cheng B, et al. Socioeconomic Deprivation Index Is Associated With Psychiatric Disorders: An Observational and Genome-wide Gene-by-Environment Interaction Analysis in the UK Biobank Cohort. Biol Psychiatry. 2021;89(9):888–95. doi: 10.1016/j.biopsych.2020.11.019 33500177

