# Supplementary material for: Non-linear association between life’s essential 8 score and depression in middle-aged and older adults with chronic obstructive pulmonary disease
Source: PLoS One. 2025 Jul 28;20(7):e0327877. doi: 10.1371/journal.pone.0327877 (PMC12303270; doi:10.1371/journal.pone.0327877)
Supplement: S1 Table — (DOCX) [file pone.0327877.s001.docx]

Table S1. Definition and scoring approach for the American Heart Association’s Life’s Essential 8 score.

| Domain | CVH Metric | Measurement | Quantification and Scoring of CVH Metric |
| --- | --- | --- | --- |
| Health  Behaviors | Diet | Healthy Eating Index-2015 diet  score percentile | Quantiles of DASH-style diet adherence or HEI-2015  (population)  Scoring (Population):  Points Quantile  100: ≥ 95th percentile (top/ideal diet)  80: 75th - 94th percentile  50: 50th -74th percentile  25: 25th -49th percentile  0: 1st -24th percentile (bottom/least ideal quartile) |
|  | Physical activity | Self-reported minutes of moderate  or vigorous physical activity per week | Metric: Minutes of moderate (or greater) intensity activity per week  Scoring:  Points Minutes  100: ≥ 150  90: 120 - 149  80: 90 - 119  60: 60 – 89  40: 30 - 59  20: 1 - 29  0: 0 |
|  | Nicotine exposure | Self-reported use of cigarettes or inhaled nicotine- delivery system | Metric: Combustible tobacco use and/or inhaled NDS use; or second hand smoke exposure  Scoring:  Points Status  100: Never smoker  75: Former smoker, quit ≥ 5 y  50: Former smoker, quit 1 - < 5 y  25: Former smoker, quit < 1 y, or currently using inhaled NDS  0 Current smoker  Subtract 20 points (unless score is 0) for living with active indoor smoker in home |
|  | Sleep health | Self-reported average hours of sleep per night | Metric: Average hours of sleep per night  Scoring:  Points Level  100: 7 - < 9  90: 9 - < 10  70: 6 - < 7  40: 5 - < 6 or ≥ 10  20: 4 - < 5  0: < 4 |
| Health Factors | Body mass index | Body weight (kg) divided by height squared(m^2^) | Metric: Body mass index (kg/m2 )  Scoring:  Points Level  100: < 25  70: 25.0 - 29.9  30: 30.0 - 34.9  15: 35.0 - 39.9  0: ≥ 40.0 |
|  | Blood lipids | Plasma total and HDL-cholesterol with calculation of non-HDL cholesterol | Metric: Non-HDL-cholesterol (mg/dL)  Scoring:  Points Level  100: <130  60: 130 - 159  40: 160 - 189  20: 190 - 219  0: ≥ 220  If drug-treated level, subtract 20 points |
|  | Blood glucose | Fasting blood glucose or casual hemoglobin A1c | Metric: Fasting blood glucose (mg/dL) or Hemoglobin A1c (%)  Scoring:  Points Level  100: No history of diabetes and FBG < 100 (or HbA1c < 5.7)  60: No diabetes and FBG 100 - 125 (or HbA1c 5.7 - 6.4) (Pre-diabetes)  40: Diabetes with HbA1c < 7.0  30: Diabetes with HbA1c 7.0 - 7.9  20: Diabetes with HbA1c 8.0 - 8.9  10: Diabetes with Hb A1c 9.0 - 9.9  0: Diabetes with HbA1c ≥ 10.0 |
|  | Blood pressure | Appropriately measured systolic and diastolic blood pressure | Metric: Systolic and diastolic blood pressure (mm Hg)  Scoring:  Points Level  100: < 120/< 80 (Optimal)  75: 120 - 129/< 80 (Elevated)  50: 130 - 139 or 80 - 89 (Stage I HTN)  25: 140 - 159 or 90 - 99  0: ≥ 160 or ≥ 100  Subtract 20 points if treated level |

CVH, cardiovascular health; FBG, Fasting blood glucose; HbA1c, Hemoglobin A1c
