# Supplementary material for: Non-linear association between life’s essential 8 score and depression in middle-aged and older adults with chronic obstructive pulmonary disease
Source: PLoS One. 2025 Jul 28;20(7):e0327877. doi: 10.1371/journal.pone.0327877 (PMC12303270; doi:10.1371/journal.pone.0327877)
Supplement: S2 Table — (DOCX) [file pone.0327877.s002.docx]

Table S2. Healthy Eating Index-2015 Components & Scoring Standards*

| Component | Maximum points | Standard for maximum score | Standard for minimum score of zero |
| --- | --- | --- | --- |
| Adequacyt | | | |
| Total Fruits | 5 | ≥0.8 cup equiv. per 1,000 kcal | No Fruit |
| Whole Fruits | 5 | ≥0.4 cup equiv. per 1,000 kcal | No Whole Fruit |
| Total Vegetables | 5 | ≥1.1 cup equiv. per 1,000 kcal | No Vegetables |
| Greens and Beans | 5 | ≥0.2 cup equiv. per 1,000 kcal | No Dark Green Vegetables or Legumes |
| Whole Grains | 10 | ≥1.5 oz equiv. per 1,000 kcal | No Whole Grains |
| Dairy | 10 | ≥1.3 cup equiv. per 1,000 kcal | No Dairy |
| Total Protein Foods | 5 | ≥2.5 oz equiv. per 1,000 kcal | No Protein Foods |
| Seafood and Plant Proteins | 5 | ≥0.8 oz equiv. per 1,000 kcal | No Seafood or Plant Proteins |
| Fatty Acids‡ | 10 | (PUFAs + MUFAs)/SFAs ≥2.5 | (PUFAs + MUFAs)/SFAs≤1.2 |
| Moderations | | | |
| Refined Grains | 10 | ≤1.8 oz equiv. per 1,000 kcal | ≥4.3 oz equiv. per 1,000 kcal |
| Sodium | 10 | ≤1.1 gram per 1,000 kcal | ≥2.0 grams per 1,000 kcal |
| Added Sugars | 10 | ≤6.5% of energy | ≥26% of energy |
| Saturated Fats | 10 | ≤8% of energy | ≥16% of energy |

*Intakes between the minimum and maximum standards are scored proportionately.

†Adequacy components represent the food groups, subgroups, and dietary elements that are encouraged. For these components, higher scores reflect higher intakes, because higher intakes are desirable.

‡Ratio of poly- and monounsaturated fatty acids (PUFAs and MUFAs) to saturated fatty acids (SFAs).

§Moderation components represent the food groups and dietary elements for which there are recommended limits to consumption. For moderation components, higher scores reflect lower intakes, because lower intakes are more desirable.
