# Supplementary material for: Non-linear association between life’s essential 8 score and depression in middle-aged and older adults with chronic obstructive pulmonary disease
Source: PLoS One. 2025 Jul 28;20(7):e0327877. doi: 10.1371/journal.pone.0327877 (PMC12303270; doi:10.1371/journal.pone.0327877)
Supplement: S3 Table — (DOCX) [file pone.0327877.s003.docx]

Table S3. Association between Life’s Essential 8 score and depression by quartile

| Variable | Crude model | |  | Model 1 | |  | Model 2 | |
| --- | --- | --- | --- | --- | --- | --- | --- | --- |
|  | OR (95%CI) | p |  | OR (95%CI) | p |  | OR (95%CI) | p |
| Total LE8 score |  |  |  |  |  |  |  |  |
| Quartile 1 | Reference |  |  | Reference |  |  | Reference |  |
| Quartile 2 | 0.87(0.54, 1.40) | 0.55 |  | 0.93(0.55, 1.57) | 0.784 |  | 1.1(0.62, 1.95) | 0.734 |
| Quartile 3 | 0.39(0.22, 0.70) | 0.002 |  | 0.4(0.21, 0.75) | 0.005 |  | 0.62(0.33, 1.18) | 0.143 |
| Quartile *4* | 0.1(0.05, 0.19) | <0.001 |  | 0.1(0.05, 0.18) | <0.001 |  | 0.18(0.09, 0.38) | <0.001 |
| *P* for trend |  | <0.001 |  |  | <0.001 |  |  | <0.001 |

Abbreviations: CI, confidence interval; OR, odds ratio; PIR, poverty income ratio, CVD, cardiovascular disease; CKD, chronic kidney disease.

Crude model: unadjusted.

Model 1: adjusted for age, sex, race/ethnicity

Model2: adjusted for age, sex, race/ethnicity, marital status, educational level, PIR, CVD history, and CKD history.
