# Supplementary material for: Non-linear association between life’s essential 8 score and depression in middle-aged and older adults with chronic obstructive pulmonary disease
Source: PLoS One. 2025 Jul 28;20(7):e0327877. doi: 10.1371/journal.pone.0327877 (PMC12303270; doi:10.1371/journal.pone.0327877)
Supplement: S4 Table — (DOCX) [file pone.0327877.s004.docx]

Table S4. Unweighted logistic analysis of association between Life’s Essential 8 score and depression

|  | Crude model | |  | Model 1 | |  | Model 2 | |
| --- | --- | --- | --- | --- | --- | --- | --- | --- |
| Variable | OR (95%CI) | p |  | OR (95%CI) | p |  | OR (95%CI) | p |
| LE8 score |  |  |  |  |  |  |  |  |
| Low (0–49) | 1(Ref) |  |  | 1(Ref) |  |  | 1(Ref) |  |
| Moderate (50–79) | 0.42 (0.3~0.58) | <0.001 |  | 0.42 (0.3~0.59) | <0.001 |  | 0.59 (0.41~0.85) | 0.005 |
| High (80–100) | 0.15 (0.05~0.5) | 0.002 |  | 0.16 (0.05~0.53) | 0.003 |  | 0.36 (0.1~1.27) | 0.113 |
| *p* for trend |  | <0.001 |  |  | <0.001 |  |  | 0.003 |
| Per 10-points increase | 0.63 (0.55~0.71) | <0.001 |  | 0.62 (0.55~0.71) | <0.001 |  | 0.72 (0.62~0.83) | <0.001 |

Abbreviations: CI, confidence interval; OR, odds ratio; PIR, poverty income ratio, CVD, cardiovascular disease; CKD, chronic kidney disease.

Crude model: unadjusted.

Model 1: adjusted for age, sex, race/ethnicity

Model2: adjusted for age, sex, race/ethnicity, marital status, educational level, PIR, CVD history, and CKD history.
