# Supplementary material for: Non-linear association between life’s essential 8 score and depression in middle-aged and older adults with chronic obstructive pulmonary disease
Source: PLoS One. 2025 Jul 28;20(7):e0327877. doi: 10.1371/journal.pone.0327877 (PMC12303270; doi:10.1371/journal.pone.0327877)
Supplement: S5 Table — (DOCX) [file pone.0327877.s005.docx]

Table S5. The relationship between LE8 and depression after imputation of missing values.

|  | Crude model | |  | Model 1 | |  | Model 2 | |
| --- | --- | --- | --- | --- | --- | --- | --- | --- |
| Variable | OR (95%CI) | p |  | OR (95%CI) | p |  | OR (95%CI) | p |
| LE8 score |  |  |  |  |  |  |  |  |
| Low (0–49) | 1(Ref) |  |  | 1(Ref) |  |  | 1(Ref) |  |
| Moderate (50–79) | 0.39 (0.28~0.54) | <0.001 |  | 0.39 (0.28~0.55) | <0.001 |  | 0.53 (0.37~0.55) | ＜0.001 |
| High (80–100) | 0.14 (0.04~0.44) | 0.001 |  | 0.13 (0.04~0.45) | 0.001 |  | 0.28 (0.08~0.97) | 0.044 |
| *p* for trend |  | <0.001 |  |  | <0.001 |  |  | 0.003 |
| Per 10-points increase | 0.62 (0.55~0.70) | <0.001 |  | 0.61 (0.54~0.70) | <0.001 |  | 0.69 (0.60~0.79) | <0.001 |

Abbreviations: CI, confidence interval; OR, odds ratio; PIR, poverty income ratio, CVD, cardiovascular disease; CKD, chronic kidney disease.

Crude model: unadjusted.

Model 1: adjusted for age, sex, race/ethnicity

Model2: adjusted for age, sex, race/ethnicity, marital status, educational level, PIR, CVD history, and CKD history.
